# Supplementary material for: Coxsackievirus A7 and Enterovirus A71 Significantly Reduce SARS-CoV-2 Infection in Cell and Animal Models
Source: Viruses. 2024 Jun 4;16(6):909. doi: 10.3390/v16060909 (PMC11209502; doi:10.3390/v16060909)
Supplement: Supplementary file 1 [file viruses-16-00909-s001.zip › Svyatc_Figure S1.pdf]

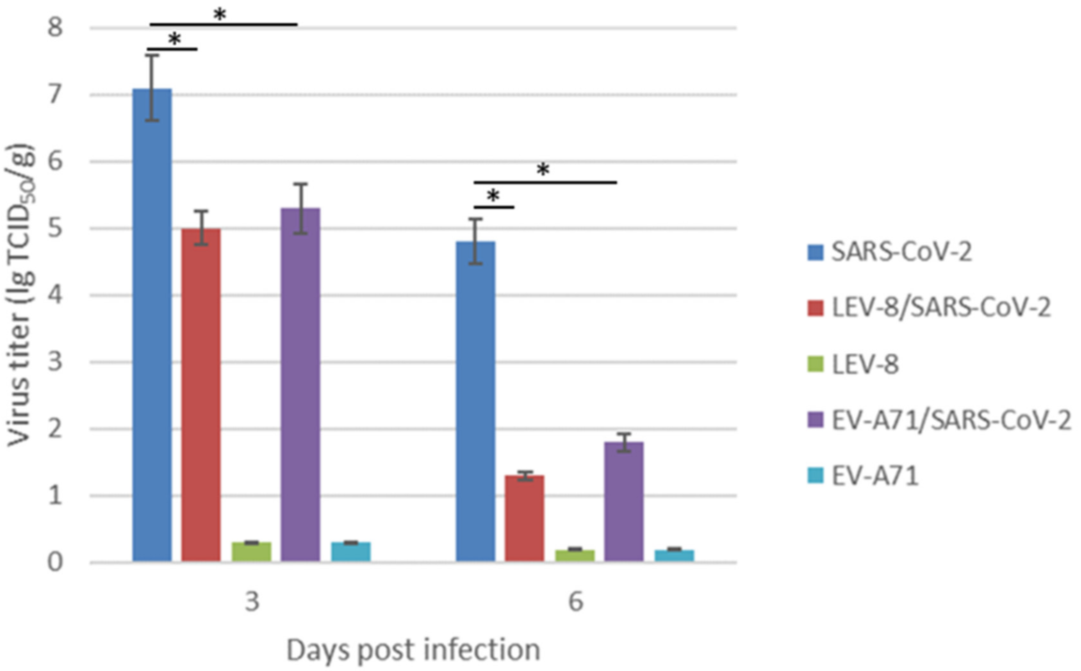

Figure S1. SARS-CoV-2 replication in Syrian hamster mono-infected and co-infected with enteroviruses.

Syrian hamsters were intranasally challenged on Day 0 for mono-infection: SARS-CoV-2 (10^5 $TCID_{50}$); LEV-8 (10^6 $TCID_{50}$); EV-A71 (10^6 $TCID_{50}$) and for co-infection: LEV-8 (10^6 $TCID_{50}$)/SARS-CoV-2 (10^5 $TCID_{50}$); EV-A71 (10^6 $TCID_{50}$)/SARS-CoV-2 (10^5 $TCID_{50}$). On Days 3 and 6 post-infection, the lung tissues collected from Syrian hamsters were homogenized to determine the viral infectious titers. The SARS-CoV-2, LEV-8 and EV-A71 titers, expressed as the 50% tissue culture infectious doses per gram ($TCID_{50}$/g), were determined by the CPE assay in Vero E6 (SARS-CoV-2) and HEK293A (LEV-8 and EV-A71) cells, respectively. The values represent the means ± SDs of three animals. Student's t-test was used for two-group comparisons. *$p < 0.05$.
