# Supplementary material for: Coxsackievirus A7 and Enterovirus A71 Significantly Reduce SARS-CoV-2 Infection in Cell and Animal Models
Source: Viruses. 2024 Jun 4;16(6):909. doi: 10.3390/v16060909 (PMC11209502; doi:10.3390/v16060909)
Supplement: Supplementary file 1 [file viruses-16-00909-s001.zip › Svyatc_Table-S1.pdf]

Table S1. Modeling the *in vitro* co-infection with LEV-8 or EV-71 and SARS-CoV-2 during simultaneous (A) and consecutive (B) infections in Vero E6 cells (viral RNA load).

(A)

	Mono-infection		Co-infection
	**SARS-CoV-2**	**LEV-8**	**SARS-CoV-2 + LEV-8**
Time post-infection	Viral RNA load, lg genome copies/mL	Viral RNA load, lg genome copies/mL	Viral RNA load, SARS-CoV-2/ LEV-8, lg genome copies/mL
24 h	6.6±0.4	7.0±0.3	4.6±0.3*/5.6±0.3*
48 h	8.4±0.3	8.6±0.4	5.5±0.3*/7.3±0.3*
	**SARS-CoV-2**	**EV-A71**	**SARS-CoV-2 + EV-A71**
24 h	7.0±0.4	7.4±0.3	4.3±0.3*/5.4±0.3*
48 h	8.5±0.3	9.0±0.4	5.3±0.3*/7.1±0.3*

(B)

Time post-infection with SARS-CoV-2	Viral RNA load, SARS-CoV-2/LEV-8, lg genome copies/mL	Viral RNA load, lg genome copies/mL
	**LEV-8 pre-infection -24h- SARS-CoV-2**	**Mock pre-infection -24h- SARS-CoV-2**
24 h	4.4±0.3*/8.2±0.4	7.7±0.3
48 h	4.8±0.4*/9.1±0.3	9.3±0.4
	**SARS-CoV-2 pre-infection -24h- LEV-8**	**Mock pre-infection -24h- LEV-8**
48 h	8.0±0.4/7.8±0.3 [n]	7.2±0.3
72 h	6.9±0.3/7.4±0.4*	9.2±0.3
Time post-infection with SARS-CoV-2	Viral RNA load, SARS-CoV-2/EV-A71, lg genome copies/mL	Viral RNA load, lg genome copies/mL
	**EV-A71 pre-infection -24h- SARS-CoV-2**	**Mock pre-infection -24h- SARS-CoV-2**
24 h	4.7±0.3*/6.5±0.2	7.3±0.3
48 h	5.0±0.4*/8.2±0.3	8.7±0.4
	**SARS-CoV-2 pre-infection -24h- EV-A71**	**Mock pre-infection -24h- EV-A71**
48 h	7.6±0.3/7.1±0.3*	8.3±0.4
72 h	7.0±0.3/7.2±0.4*	8.0±0.3

Note: Values represent means ± SD of three independent experiments. Student's t-test was used for two-group comparisons, *- $p< 0.05$, [n]—not statistically
