# Supplementary material for: Coxsackievirus A7 and Enterovirus A71 Significantly Reduce SARS-CoV-2 Infection in Cell and Animal Models
Source: Viruses. 2024 Jun 4;16(6):909. doi: 10.3390/v16060909 (PMC11209502; doi:10.3390/v16060909)
Supplement: Supplementary file 1 [file viruses-16-00909-s001.zip › Svyatc_Table-S2.pdf]

Table S2. Viral titers in harvested tissues from Syrian hamster mono-infected and co-infected with SARS-CoV-2, LEV-8 or EV-A71.

**Tissue**	**SARS-CoV-2**	**LEV-8**	**EV-A71**	**LEV-8/3 days/SARS-CoV-2**	**EV-A71/3 days/SARS-CoV-2**
	lg $TCID_{50}$	lg $TCID_{50}$	lg $TCID_{50}$	lg $TCID_{50}$ (SARS-CoV-2/LEV-8)	lg $TCID_{50}$ (SARS-CoV-2/EV-A71)
Brain	<1.0	<1.0	<1.0	<1.0/<1.0	<1.0/<1.0
Intestine	1.7±0.2	2.8±0.3	3.1±0.3	<1.0/2.6±0.3	1.0±0.2/3.0±0.3
Feces	1.4±0.2	2.3±0.2	2.9±0.3	<1.0/2.0±0.2	<1.0/2.5±0.3

Virus titer is expressed as the mean lg $TCID_{50}$ ± standard error of mean per 10% tissue homogenates derived from 3 hamsters at day 3 post infection with SARS-CoV-2 (at day 6 post infection with LEV-8 or EV-A71).
