# Supplementary material for: Coxsackievirus A7 and Enterovirus A71 Significantly Reduce SARS-CoV-2 Infection in Cell and Animal Models
Source: Viruses. 2024 Jun 4;16(6):909. doi: 10.3390/v16060909 (PMC11209502; doi:10.3390/v16060909)
Supplement: Supplementary file 1 [file viruses-16-00909-s001.zip › Svyatc_Table-S3.pdf]

Table S3. Evaluation of lung pathology for infected Syrian hamsters

The pathological lesions	Control		SARS-CoV-2			LEV-8			LEV-8/3day/SARS-CoV-2		
Interstitial pneumonia involving neutrophils and heterophils	0	0	2	3	3	0	0	0	1	1	1
Acute diffuse alveolar damage, necrosis of alveolar epithelial cells	0	0	2	3	3	0	0	0	0	0	0
Bronchitis with necrosis of bronchial epithelial cells	0	0	2	2	3	0	0	0	0	1	0
Hyperplasia of the bronchial epithelium	0	0	1	2	1	0	0	0	0	1	0
Endotheliitis with necrosis and desquamation of endothelial cells	0	0	1	1	2	0	0	0	0	1	0
Perivascular edema and perivascular lymphocytic infiltration	0	0	2	3	2	0	0	0	0	1	0
Intraalveolar hemorrhages	0	0	2	2	2	0	0	0	0	0	0

Note. The pathological changes in the lung according to the scale: 0 – no pathology; 1– mild lesions; 2 – moderate lesions; 3 – pronounced lesions [1]. Mock-infected (2 animals), SARS-CoV-2, LEV-8, LEV-8/3 day/ SARS-CoV-2 (3 animals).

Three evenly distributed sections from each lung were scored for various parameters.

1. Gruber, A.D.; Osterrieder, N.; Bertzbach, L.D.; Vladimirova, D.; Greuel, S.; Ihlow, J.; Horst, D.; Trimpert, J.; Dietert, K. Standardization of Reporting Criteria for Lung Pathology in SARS-CoV-2-infected Hamsters: What Matters? *Am J Respir Cell Mol Biol*. **2020,** 63(6), 856-859. https://doi.org 10.1165/rcmb.2020-0280LE.
